# Medical News and Miscellany

**Published:** 1897-03

**Authors:** 


### Medical News and Miscellany.

**Dr. O. M. Conerly** has removed from El Paso to Atlanta, Texas, where he will do a general practice.

**Dr. D. L. Peeples,** of Navasota, is taking a special course at the St. Louis Post Graduate Medical College.

**Dr. W. R. Blackshear** has removed from Brookland, Sabine county, to Chireno, Nacogdoches county, Texas.

**Wanted**—To buy a home with a practice on a railroad in Central or Southern Texas. Address Dr. Edwards, Paris, Texas.

**The trustees** of the New York Polyclinic Medical School and Hospital have decided to rebuild on the site of their former building, Nos. 214, 216 and 218 E. 34th st. The work will begin immediately.

**Dr. Vard H. Hulen.**—The co-partnership previously existing between Drs. Hall and Hulen, of Galveston, has been dissolved by mutual consent, each partner continuing the practice on his own account; that of diseases of the eye, ear, nose and throat.

**Asylum Changes.**—Dr. Jno. S. Turner, of Granberry, Hood county, has been appointed assistant physician to the Southwest Texas Insane Asylum at San Antonio, vice Dr. T. T. Jackson, resigned. Dr. Jackson has located at San Antonio to engage in the general practice.

**The Epileptics.**—The Board of State Commissions of Public Charities of Illinois has recommended in their report that the State provide a separate institution for epileptics, and states that there is one epileptic to each six hundred population, or about 8000 epileptics in that State. What has become of the Texas State Medical Association memorial on this subject?

---

**The Laryngoscope,** published in St. Louis, has been selected as the official organ, for the year 1897, of the Laryngological Section of the New York Academy of Medicine. This selection, and the great probability of the same journal being chosen by other Laryngological, Rhinological and Otological societies as their official organ, would indicate that *The Laryngoscope* has become what its proprietors stated they intended to make it, i. e., the American journal of record for the specialties represented.

---

**The appeal** to the JOURNAL for advice on the part of one who signs himself "An Unfortunate Young Man"—has elicited quite an outpouring of that commodity, as will be seen by reference to our Correspondence Department,—not less than five physicians having contributed their views of treatment. It ranges all the way from hypnotism to surgical procedure, and embraces various lines of therapeusis. Taken altogether it constitutes a pretty thorough discussion of the treatment of the condition resulting from that folly for which God cursed one Onan in olden times. We adhere still to our own remedy,—others failing; if the *testicle* offend, pluck it out. In a multitude of council there is much wisdom. The young man can take his choice here of several plans.

---

**Our subscribers** are respectfully reminded that it takes money, ready money, with each issue, and lots of it, to get out such a red hot, bilin' publication as the "Red Back"; acknowledged to be the most popular, influential, progressive, independent and original of its kind, and that remittances are *always* in order. One fellow thinks that everybody else is remitting, and that his little two dollars will not be missed. But, too many think just as he does, and hence. Send along your $2 to Uncle Daniel; he needs it. If you can't send all you owe—and you, *every one* know how you stand, for I have billed you, just feel in your right hand vest pocket and send what you can spare, if it is only a $2 bill. We are not running this JOURNAL for fun, tho' there is a good deal of fun in it; see?

**The** *New York Medical Record* complains that the anti-spitting law in New York city is not enforced. On the street cars the conductor is entrusted with its enforcement, and the *Record* says when a complaint is made to a conductor he has to empty his mouth of a deluge of tobacco juice preliminary to remarking, "Shucks, that law ain't nothin.'"

---

**A good practice** in six miles of one of the best middle-sized cities in Texas can be secured on favorable terms by applying to the JOURNAL. Country practice, in a thickly settled German settlement,—most of them speak English,—and usually have the cash on hand to pay the doctor. Nets $1800 to $2400. This practice can be had by buying the incumbent's property; it consists of three acres of land, a five-room cottage and office—a good barn and other outbuildings. $2000, half cash; the incumbent will induct purchaser into practice and retire at any time.

---

**Getting in Their Work.**—In April, '95, the law against quacks went into effect in New York. Since which time the New York County Medical Society has caused the arrest of eighty-three persons for illegal practice of medicine, and has obtained convictions in fifty-one cases. These fifty-one persons have paid $3,690 in fines, and a number of them have been sent to jail besides. The legal department of the Society has a large number of cases pending, says the New York *Medical Record.* Why cannot the Texas State Medical Society have a legal department to look after these ducks?

---

**Quarantine Appointments.**—The quarantine officers and inspectors under the last administration have been re-appointed: They are as follows:

Dr. R. M. Swearingen, State Health Officer.
Dr. J. C. Mayfield, Quarantine Officer, Galveston.*
Dr. A. N. Perkins, Quarantine Officer, Sabine Pass.
Dr. T. J. McFarland, Quarantine Officer, Pass Cavaels.
Dr. W. E. Pugh, Quarantine Officer, Rockport.
Dr. A. S. Wolff, Quarantine Officer, Brownsville.
Dr. E. S. Weisigar, Quarantine Officer, Velasco.
Dr. W. M. Yandell, Quarantine Inspector, El Paso.
Dr. T. J. Turpen, Quarantine Inspector, Laredo.
Dr. A. H. Evans, Quarantine Inspector, Eagle Pass.

---

*Appointed in November, 1896, vice Dr. Blunt, who resigned before the close of the term of his appointment.

**Dr. W. L. York,** of Decatur., Tex., has gone to Mineral Wells, Tex., where he has formed a copartnership in the practice with Dr. J. H. McCracken. The doctor's host of friends all over the South will be pleased to hear that his health, which for some time past has been impaired, is measurably restored, and it is hoped that a sojourn at Mineral Wells and the use of those splendid waters will restore him to his former vigor and strength. Let them remember that while "resting" the doctor wants to be at work; keeping his hand in at least, and when any of the doctor's friends find it advisable to send a patient to the Wells—consign him to the care of York and he will have conscientious care and attention. The profession of Texas can not spare York yet. We hope he has definitely given out all idea of dying, at least for many years.

---

**Kind Words.**—The *Red Back* is much gratified at the numerous kindly expressions of appreciation on the part of its readers, which are spontaneous and heartfelt. They tell the tale. The JOURNAL is sowing seed, many of which are falling in good ground and are producing fruits: a missionary work, which is being felt. In a recent mail we received the following high testimonial, which speaks for itself:

"Yours is comparatively a high-priced journal, but I have ever appreciated it, especially for your caustic criticism of charlatans and impostors, and your effort to elevate the standard of the medidal profession.

"I am an old physician, now in my fiftieth year of continuous practice; still, a live journal like yours is of inestimable value to me, as I find in it many practical hints and scientific deductions, collated from the medical world, that no one M. D.'s experience can cover.

Leesville, Texas. "C. MCGARITY, M. D."

---

**Science Gone to Seed.**—There is but one step from the sublime to the ridiculous, and it is often a very short one. This step is taken unconsciously, sometimes, and it then becomes amusing. Scientific investigation is commendable when carried on with a view of ascertaining truths that have a bearing on human progress and happiness; but when costly experiments are conducted by alleged scientists that can result in no possible good, it is a rape on science; her fair name is taken in vain. What possible good can come from tying a vein, or the thoracic duct in a lot of rabbits, for instance, and then dosing them with alcohol, and taking the temperature? These experiments are given with the most minute detail, in a big new journal called the "Journal of Experimental Research" ($5 a year!). But the climax of absurdity is reached in an article in a late issue of that publication, by Professors Kennedy and McGrath, "On the Relation of the Volume of the Coronary Circulation to the Frequency and Force of the Ventricular Contractions of the Isolated Heart of a *Cat!*" (Color and kind of cat not stated, nor whether of the Thomas or the Tabitha peasuasion.)

---

**A Lotion for Acne Punctata.**--Dr. A. Malbec (*Province Medicale*, November 28, 1896) recommends this formula:

℞ Borax,<br>

Sodium bicarbonate, } each......10 parts.<br>

Ether..................................20 parts.<br>

Rose water............................300 parts.

M. To be used after pressing out the contents of the follicles and in conjunction with frictions twice a day with sulphur soap and very hot water.

---

**The Plague.**—The *Journal American Medical Asssociation* says: "The French government has appointed Dr. Yersin, of Paris, to proceed to India and use his anti-plague serum. The French do not expect Dr. Yersin to conduct his investigations by letter. That method is peculiar to Washington. Let us have a department of "Public Health." Amen.

The *New York Medical Record* says:

"Up to February 4th, according to the official returns, there had been in Bombay 5,098 cases of the plague, with 3,841 deaths. Several of the European governments are sending commissions to India to study the disease. It was announced that Koch was to head the one sent by Germany, but a dispatch from Berlin says that he has telegraphed from South Africa his refusal to go to Bombay. Yersin is now on his way there, having been invited by the government to make a trial of his antiplague serum, which was found so effectual in Amoy last year. A sanitary conference has been called to meet in Venice on February 12th. It is announced that the British delegates will refuse to sanction the adoption of stringent quarantine restrictions, urging rather upon the delegates from Southern Europe the prophylactic virtues of cleanliness, both personal and municipal. It is announced by the governor of the Transcaspian territories that the bubonic plague has appeared at Candahar, Afghanistan. A force of Cossacks has been sent to the Amu Darya River to prevent, if possible, the introduction of the disease into Russian territory."

Yersin went to China, where by bribing guards he was enabled to obtain pus from the bubos; this he inoculated into horses and obtained his serum. Kitassito, the Japanese physician is credited with first demonstrating the bacillus of plague.

